# Effect of Using Different Kinds and Ratios of Vegetable Oils on Ice Cream Quality Characteristics

**DOI:** 10.3390/foods7070104

**Published:** 2018-07-03

**Authors:** Mehmet Güven, Murat Kalender, Tansu Taşpinar

**Affiliations:** Faculty of Agriculture, Food Engineering Department, Cukurova University, Adana 01330, Turkey; mguven@cu.edu.tr (M.G.); mkalender@cu.edu.tr (M.K.)

**Keywords:** ice cream, vegetable oil, olive oil, hazelnut oil, physicochemical and sensory properties

## Abstract

The aim of this study was to develop ice cream products using different types of oils, a sensory ballot to focus on the textural attributes of new ice cream products, evaluate physicochemical properties of these products and physical measurements. Milkfat, hazelnut oil and olive oil were mixed at different concentrations for a total of 12% fat. Control sample contains 12% milk fat while the other formulations contain different proportion of milk fat, hazelnut oil and olive oil as the fat content. The combination of the different proportion of milk fat, hazelnut oil and olive oil are given as % milk fat, % hazelnut oil and % olive oil respectively; 12:0:0, 0:12:0, 0:0:12, 6:6:0, 6:0:6, 0:6:6, 4:4:4. The pH, free acidity, total solid ingredient, b* value and volume increase rate were statistically significant (*p* < 0.05). Sensory analysis results showed that: samples were 50% hazelnut oil-50% olive oil had the highest color and appearance scores. On the other hand, the highest score in body and texture scores were belongs to the sample of used 50% milk fat-50% hazelnut oil and 50% milk fat-50% olive oil, 50% milk fat-50% olive oil the most preferred ones in total quality criterions.

## 1. Introduction

Ice cream is a frozen product consumed all over the world in dairy products [1]. After the first and general definition, ice creams have been defined as food systems called polyphasic. These daily products include ice crystals, air bubbles, protein-hydrocolloid structures, a cryoconcentrated aqueous phase, emulsified fat, proteins and salts. In this regard, ice creams may be evaluated as oil-in-water emulsions [2].

In ice cream, fat and fat structure development tissue has a critical prescription for many features. These properties include stability, optimal structure, physical properties as an example [3]. Also, fat network governs many desirable quality properties. It produces a smooth characteristic, increases the richness of flavor in ice cream, a good carrier and synergist for added flavor compounds, helps to stabilize the foam, largely responsible for the creamy texture, contributes good melting properties, helps to provide ice cream structure, aids in lubricating the freezer barrel while the ice cream is being frozen [4].

With the reason of increased consumption of reduced or low fat dairy products, producers have made some changes including regulating product proportions that is unsaturated or “healthier” fats [5].

Replacing the currently used solid fat with “healthier” or liquid fractions is a major challenge. Research on the physicochemical properties of ice cream incorporated with both of which are used hazelnut oil and olive oil have not been shown up till today. The objectives of this study was to develop ice cream products using different types of oils, a sensory ballot to focus on the textural attributes of new ice cream products, to evaluate physicochemical properties of these products and physical measurements, evaluating the changes that occur in an ice cream formulation made with different kinds and proportions of milk fat, hazelnut oil and olive oil.

The finding will contribute to our understanding of the physicochemical properties, structures and texture associated with different oil used ice creams.

## 2. Materials and Methods

### 2.1. Materials and Mix Preparation

To product the ice cream, some materials were included; milk, skimmed milk powder (SMP), milk fat, vegetable oil (hazelnut oil and olive oil), sugar and the mixture of emulsifier and stabilizer.

All ice creams consisted of 5% skim milk powder, 13.5% sucrose, 3.3% stabilizer and emulsifier blend. Milk, skim milk powder and milk fat were obtained from Cukurova University Agricultural Faculty Research and Application Farm. Sucrose, hazelnut oil and olive oil were purchased from a local market (Adana, Turkey). Used hazelnut oil is brand of Fiskobirlik and it contains 34 mg of vitamins in 100 g. The energy and nutrients found in 100 g are as follows; energy: 900 kcal, protein 0 g, fat 100 g (saturated fatty acids: 7.6 g, trans fatty acids 0 mg, monounsaturated fatty acids 80.6 g, polyunsaturated fatty acids 11.8 g, cholesterol 0 g, carbonhidrat 0 g, tocoferol 34 mg). Used olive oil is brand of Tariş. The energy and nutrients found in 100 g are as follows; energy: 900 kcal, protein 0 g, fat 100 g (saturated fatty acids: 15.5 g, monounsaturated fatty acids 74 g, polyunsaturated fatty acids 10.5 g, total carbohydrate 0 g, protein 0 g, salt 0 g, minerals 0 g). Stabilizer and emulsifier blend was obtained from Sigma.

Milk fat, hazelnut oil and olive oil were mixed at different concentrations for a total of 12% fat. Six ice cream formulations and one control were designed. Control sample contains 12% milk fat while the other formulations contain different proportion of milk fat, hazelnut oil and olive oil as the fat content. The mixture of the different proportion of milk fat, hazelnut oil and olive oil are given as milk fat %, hazelnut oil % and olive oil % respectively; 12:0:0, 0:12:0, 0:0:12, 6:6:0, 6:0:6, 0:6:6, 4:4:4.

### 2.2. Ice Cream Production

Ice cream samples were manufactured in the Dairy Technology Laboratory of Food Engineering Department, Faculty of Agriculture, University of Cukurova.

Firstly, liquid ingredients were heated up to 50 °C before the addition of solid ingredients. After milk, milk fat (samples with milk fat in it) and vegetable oils heated 50 °C skimmed milk powder, sugar and emulsifier-stabilizer blend added and the ingredients were mixed. Heating process continued until the pasteurization temperature. Ice cream mix was pasteurized at 85 °C, for 1 min. After that, with ultra-turrax prehomogenization step was carried out (Ultra-Turrax TP 18-10 IKA WERK, Berlin 65/ München 8, Germany). After prehomogenization the mixes homogenized in a 2-stage homogenizer (Heidolph DIAX 900, Munich, Germany), and then cooled to 4 °C and aged for 24 h at 4 °C to ensure complete hydration of all ingredients. Ice cream mixes were frozen using a batch freezer (Model 104; Taylor Co., Rockton, IL, USA). Target temperature was −5 °C, it was controlled by a digital thermometer that came to that temperature and until this temperature ice cream mix whipped and frozen. Ice cream samples were packaged in 100 g Styrofoam cups. After packaging ice cream samples immediately stored into a hardening room at −35 °C.

Three batches of ice cream were produced. Physicochemical and sensory characteristics were determined at 1, 45 and 90 days of the storage period. All analyzes were performed in two parallel lines and the averages of the parallel lines were taken when data is entered into the statistical program. The effects of oils and their relations on physicochemical and sensory characteristics of the ice cream were researched.

The ice cream samples were named as follows, taking into account the oil type in the ingredients; M 12% milk fat, H 12% hazelnut oil, O 12% olive oil, MH 6% milk fat-6% hazelnut oil, MO 6% milk fat-6% olive oil, HO 6% hazelnut oil-6% olive oil, MHO 4% milk fat-4% hazelnut oil-4% olive oil.

### 2.3. Basic Nutrient Analysis

Ice cream samples were analyzed for pH, total solid content, free acidity, fat and protein content.

#### 2.3.1. pH

The pH values of the ice creams were measured using a digital pH meter with a glass electrode of Testo 230 (Testo Ltd., Lenzkirch, Germany). Before measurement pH meter calibrated with pH buffer solutions (4.0, 7.0 and 10.0) and then measured at 1, 45 and 90-day storage periods [6].

#### 2.3.2. Total Solid

Total solid content of ice cream was determined according to AOAC (Association of Official Agricultural Chemists) standarts. The total solids of samples were determined by drying samples at 105 °C overnight to constant weight using an air oven [7].

#### 2.3.3. Free Acidity

Free acidity of ice cream was evaluated with titration 0.1 N of sodium hydroxide and taking 10 g of each sample adding 90 mL of distilled water with added phenolphthalein indicator and titrated until the first dye of light pink is permanent. Acidity is calculated as percentage of lactic acid (1 mL of 0.1 N NaOH = 0.09 g lactic acid) [8,9].

#### 2.3.4. Fat and Protein Content

Ice cream was analyzed for fat content and protein content by applying the Gerber method [10] and Kjeldahl method [11].

### 2.4. Overrun

Compared the weight of a fixed volume of ice cream mix and ice cream was used to define the overrun value. The formula is as follows and the results was expressed as %.

(1) $$\mathrm{Overrun}\;=\;\frac{M-I}{I}\times \;100$$

*M* = Weight of mix

*I* = Weight of ice cream [12]

### 2.5. Viscosity

Viscosity of the ice cream was measured using a Brookfield Viscometer DV-II Pro with spindle 64S for 15 and 30 s at 120 rpm and reported as cP. Ice cream was melted at 4 °C for 24 h before viscosity measurements.

### 2.6. First Dripping Time and Complete Melting Time

The mesh was placed on a ring stand suspended over a beaker. The samples were placed on this 1 mm stainless steel wire mesh (10 holes per 2.54 cm, wire thickness 0.9 mm) at ambient temperature (20 ± 1 °C) and the test was allowed to continue until all the ice cream had dripped completely through the mesh. Times at which the first drop and complete melting time observed and expressed as first drop time and complete melting time [13].

### 2.7. Colour

The color of ice creams was measured with a Minolta CR-400 colorimeter (Minolta Model CR-400, Camera Company, Osaka/JAPAN). The colorimeter has an 8 mm diameter viewing area and the measurements were recorded as L* (lightness), +a* (redness), +b* (yellowness) color co-ordinates. The coordinates L*, a* and b* were obtained using the CIE system, where L* is a measure of the lightness, a* varies from green (−) to red (+) and b* varies from blue (−) to yellow (+) using D65 illuminant and observer at 10°. The color evaluation was done in triplicate on each sample [14].

### 2.8. Sensory Evaluation

Sensory analyzes were performed by 7 trained panelists, aged 28–40 years, with four males and three females. Samples were coded with three random digit numbers and the serving order was also randomized. A 5-point hedonic scale was used which differ 1 = dislike extremely 2 = dislike moderately 3 = neither dislike nor acceptable 4 = moderately acceptable 5 = extremely acceptable. The sensory analysis parameters were color and appearance, body and texture, flavor and smell of ice cream samples. Sampling spoon, napkins, a glass of water for mouth washing, crackers, the descriptive language instructions and score cards were given to each panelist for sensory evaluation [15,16].

### 2.9. Statistical Analysis

Statistical analyses of the data were performed with SPSS 22 statistical package program. Analysis of variance was obtained with One-way analysis of variance (ANOVA) routines and multiple comparisons of means were conducted using Duncan multiple comparison test. Statistical significance is given by p values, with differences at the 95% confidence interval (*p* < 0.05) being considered statistically significant. Results are the average of three replicates for each production (SPSS Institute Inc., Cary, NC, USA).

## 3. Results

### 3.1. Milk Properties

Evaluated milk, which was used in this study, contained 8.91% of total solids, 0.75% of ash, 0.01% of fat, 4.43% of protein and titration acidity value was 0.11%. Table 1 shows results of basic nutrient composition of milk.

### 3.2. Quality Characteristics of Ice Creams

The evaluation of possible applications for new fats is based on physical properties and the solid fat content is one of the most important ones [17]. Table 2 shows total solid, ash, protein content, fat content, free acidity and pH of ice creams.

Hui et al. (2004) [18] put forward that ice creams which containing 12–15% fat has 38–40% total solid content. Sample M had a significant lowest total solid value than other samples. Also, this value was lower than the value of Hui et al. mentioned. Ash content varied between 0.90% to 1.16%. No statistically significant difference was found between the samples. It is considered that there is not a statistically significant difference because other raw materials used except for the fat type are common. Fat content of the seven ice cream samples which ranged from 11.46% to 12.0%. The results are expected as the fat ratio during the production process is set to 12%. The natural titratable acidity of the ice cream mixes depended on the ice cream MSNF percentage and could be theoretically calculated through multiplication of the milk solids not fat (MSNF) percentage by 0.017 (Goff & Hartel, 2013). Ice creams had titratable acidity of 0.17%, 0.18%, 0.19%. Statistically significant differences were found between the samples. pH value ranged between 6.61–6.64 and it was observed that the pH value of olive oil used samples increased. The results similar to the protein and pH results obtained in this study were obtained by Ullah et al. (2017) [19] which found protein results around 4% and pH results between 6.63–6.71. Fat type did not influence the ash, protein and fat content of ice creams whereas fat type caused significantly differences for other results. The percentage of total solid, free acidity and pH were found statistically different for different formulations of ice creams (*p* < 0.05).

### 3.3. Viscosity Measurement

**Figure 1:**
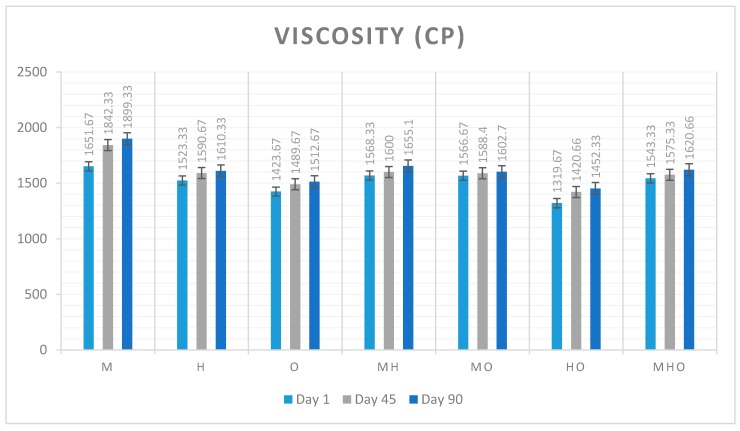
Changes in viscosity values of seven different types of ice cream samples for 15 s over three different storage days. (Abbreviations are: M 12% milk fat, H 12% hazelnut oil, O 12% olive oil, MH 6% milk fat-6% hazelnut oil, MO 6% milk fat-6% olive oil, HO 6% hazelnut oil-6% olive oil, MHO 4% milk fat-4% hazelnut oil-4% olive oil).

**Figure 2:**
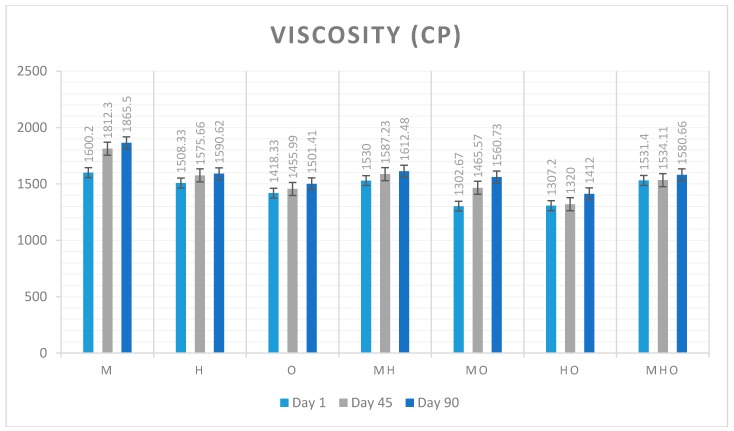
Changes in viscosity values of seven different types of ice cream samples for 30 s over three different storage days. (Abbreviations are: M 12% milk fat, H 12% hazelnut oil, O 12% olive oil, MH 6% milk fat-6% hazelnut oil, MO 6% milk fat-6% olive oil, HO 6% hazelnut oil-6% olive oil, MHO 4% milk fat-4% hazelnut oil-4% olive oil).

Apparent viscosity is a physical property of ice cream. According to Goff and Hartel (2013) viscosity is especially important for industrial design but there is not a clear optimum value for viscosity. Figure 1 shows viscosity values of seven different types of ice cream samples for 15 s over three different storage days and Figure 2 shows viscosity values of seven different types of ice cream samples for 30 s over three different storage days. In this study, for the 15 s and 30 s measured values the increased level of viscosity was the M sample made with 12% milk fat and after that MH sample made with 6% milk fat and 6% hazelnut oil. It was observed that the samples made with milk fat had more tight structure. After milk fat used samples milk fat mixed with other oils had more tight structure. The lowest viscosity occurred for the O sample made with 12% olive oil. The 15 and 30 s values were found to be close to each other. It has been observed that throughout the storage period, all samples gain a slightly tighter structure.

### 3.4. Colour

Color differences among ice creams with different kinds of fat content were observed by instrumental analysis. Table 3 shows color values of seven types of ice creams. In all samples, L* values ranged from 85.52 to 88.85, a* from −3.48 to −2.87, and b* from 8.70 to 10.46. The 4% milk fat-4% hazelnut oil-4% olive oil ice cream had a lower L* value than the other ice cream samples. The type of fat did not affect a* value as statistically but the a* value decreased with increasing level of olive oil. The b* value was found different between treatments. When the both hazelnut oil and olive oil levels increased, it was found that the b* value for the control increased accordingly. For color change scores, L* and a* values, no detectable changes were found in all seven types of the ice creams up to 3 months frozen-storage.

### 3.5. Overrun

**Figure 3:**
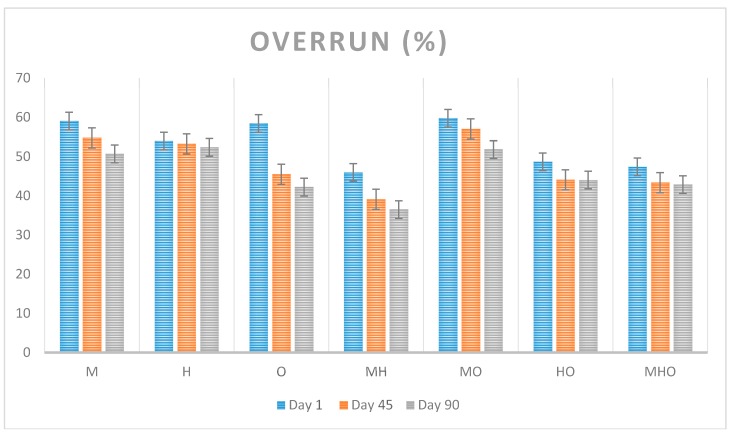
Changes in overrun values of seven different types of ice cream samples over three different storage days. (Abbreviations are: M 12% milk fat, H 12% hazelnut oil, O 12% olive oil, MH 6% milk fat-6% hazelnut oil, MO 6% milk fat-6% olive oil, HO 6% hazelnut oil-6% olive oil, MHO 4% milk fat-4% hazelnut oil-4% olive oil).

Figure 3 shows changes in overrun values of seven different types of ice cream samples over three different storage days. First day overrun values ranged from 45–59% in ice creams depending on the fat composition. Forty-fifth day overrun values ranged from 39–57% and ninetieth day overrun values ranged from 36–52%. There were significant differences between overrun values of ice creams except for the first storage day that was found between M sample made with 12% milk fat and MO sample made with 6% milk fat and 6% olive oil (*p* < 0.05). Hui et al., (2004) mentioned that ice creams which containing 12–15% fat has 60–90% overrun. The data obtained as a result of this study were found to be relatively low. Bazmi and Relkin (2009) [20] has shown enhanced foamability in milk fat ice cream when the saturated fat is partially replaced with olein-rich fractions but in this study the highest overrun was obtained in 12% milk fat ice cream at the 1th storage day whereas the lowest in 6% milk fat-6% hazelnut oil ice cream at 90th storage day (*p* < 0.05). The lower overrun values could be due to oil wetting or spreading. Statistical differences were found between the overrun values of the ice cream mixes at different storage day via one-way ANOVA (*p* < 0.05) and Duncan test.

### 3.6. First Dripping Time and Complete Melting Time

Milk fat or vegetable oils reduce the heat transfer rate through the ice cream; then, as the fat content of an ice cream increases, its melting rate decreases [21], but in this study the fat amount of all of the ice cream samples was the same and so fat type and combinations effects was searched. To evaluate the first drop time to control ice cream stability was performed.

**Figure 4:**
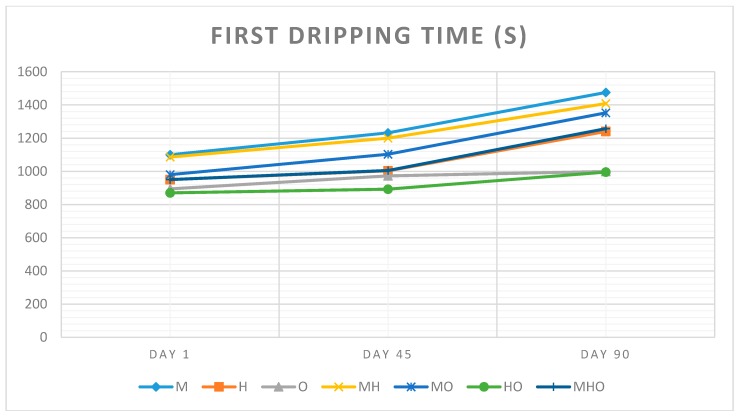
Changes in first dripping time of seven different types of ice cream samples over three different storage days. (Abbreviations are: M 12% milk fat, H 12% hazelnut oil, O 12% olive oil, MH 6% milk fat-6% hazelnut oil, MO 6% milk fat-6% olive oil, HO 6% hazelnut oil-6% olive oil, MHO 4% milk fat-4% hazelnut oil-4% olive oil).

**Figure 5:**
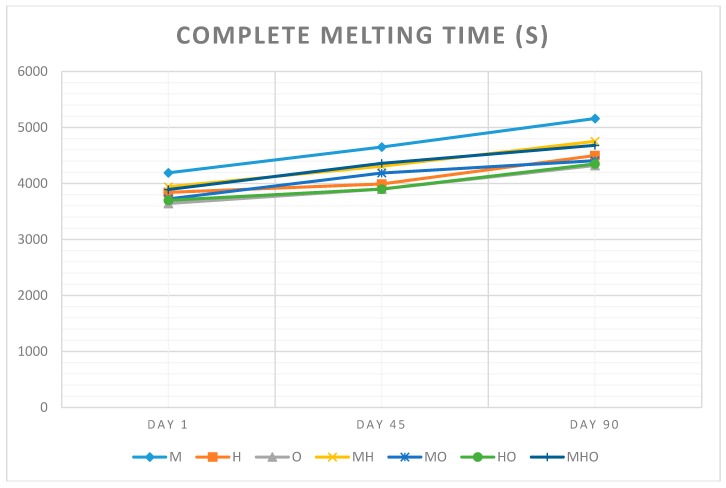
Changes in complete melting time of seven different types of ice cream samples over three different storage days. (Abbreviations are: M 12% milk fat, H 12% hazelnut oil, O 12% olive oil, MH 6% milk fat-6% hazelnut oil, MO 6% milk fat-6% olive oil, HO 6% hazelnut oil-6% olive oil, MHO 4% milk fat-4% hazelnut oil-4%olive oil).

Figure 4 and Figure 5 show changes in first dripping time and complete melting time of seven different types of ice cream samples over three different storage days. It can be said that different ratios of milk fat, hazelnut oil and olive oil affected the rates of ice cream meltdown (*p* < 0.05).

It has been showed that the time required for the first drop drained from ice cream was ranged between 894 s and 1475 s due to the meltdown results. The complete melting time was 3840 and 5161 s. The sample made with 12% milk fat formula, had the slowest melt rate with all other samples melting much faster. Based on the results on the melting rate of the ice cream samples, it was found that the when the amount of milk fat was increased the rate of meltdown decreased. The meltdown rate also decreased when the milk fat incorporated other oils. As regards the type of fat, hazelnut oil and olive oil worsened the ice cream melting. Similar results were obtained by Moriano and Alamprese (2017) [22] who studied that the possibility of producing healthier artisanal ice creams by substituting milk cream with phytosterol organogel systems based on sunflower oil.

### 3.7. Sensory Evaluation

Some of the sensory attributes of ice creams was mainly affected by fat types. Table 4 shows the results of sensory evaluation on the 7 ice cream samples for the storage period of 1, 45 and 90 day. Panelists detected no significant differences in color and appearance or body and texture among the ice creams. Milk fat contributes significantly to the rich and creamy flavor as well as firm texture of ice cream [12]. Insignificant differences of flavor scores were observed between samples M, H, MO on the other hand significant differences observed between O, MH, HO, MHO samples. The use of vegetable oils and the combination of milk fat with other vegetable oils largely affected the flavor of ice cream samples. On the basis of this research we can claim that the sample made with 12% milk fat had higher points. The O sample received relatively low scores compared to other ice cream samples. MH sample viewed low scores during two storage days. Generally, the average overall acceptability scores were close to each other but there was significant difference (*p* < 0.05) existed for the scores of total acceptance between all the ice cream samples.

## 4. Conclusions

In conclusion beside milk fat, unsaturated fats, hazelnut oil and olive oil, were successfully used for the production of ice cream. Ice creams produced with hazelnut oil or olive oil containing 12 g/100 g gelators showed similar or even better quality characteristics with respect to samples made with milk fat. It has been showed from the results obtained from here that combined oils created blends with appropriate physicochemical properties with broad applications, the application of different kinds oils in ice creams is a successful approach. The results obtained in this work, are of great interest for the production of healthier ice creams. In the current consumption market, regular fat ice cream provides a greater mouthfeel. Industrially, further research should be conducted which investigating the use of vegetable oils in ice cream for the development of a new product and validate these results with more realistic processing conditions and equipment.

## Figures and Tables

**Table 1:** Results of basic nutrient composition of milk.

	Total Solid (%)	Ash (%)	Fat (%)	Protein (%)	Titration Acidity
**Milk**	8.91 ± 0.15	0.75 ± 0.09	3.5 ± 0.00	4.43 ± 0.79	0.11 ± 0.00

**Table 2:** Results of physicochemical analysis of seven types of ice cream.

Ice Cream	Total Solid (%)	Ash (%)	Protein (%)	Fat (%)	Titration Acidity (%)	pH
M	34.40 ± 4.33 ^b^	0.90 ± 0.16 ^a^	4.76 ± 0.52 ^a^	11.81 ± 0.07 ^a^	0.17 ± 0.01 ^b^	6.61 ± 0.01 ^c^
H	41.47 ± 1.12 ^a^	1.00 ± 0.08 ^a^	5.50 ± 0.16 ^a^	11.88 ± 0.12 ^a^	0.17 ± 0.01 ^b^	6.62 ± 00 ^b,c^
O	42.06 ± 0.98 ^a^	0.97 ± 0.03 ^a^	5.18 ± 0.08 ^a^	12.00 ± 0.01 ^a^	0.18 ± 0.00 ^b^	6.63 ± 0.00 ^a,b^
MH	41.88 ± 1.33 ^a^	0.94 ± 0.08 ^a^	5.41 ± 1.70 ^a^	11.98 ± 0.02 ^a^	0.17 ± 0.07 ^b^	6.61 ± 0.01 ^c^
MO	40.53 ± 0.39 ^a^	1.02 ± 0.09 ^a^	5.63 ± 1.26 ^a^	11.83 ± 0.78 ^a^	0.18 ± 0.02 ^a,b^	6.63 ± 0.00 ^a,b^
HO	39.37 ± 2.12 ^a^	1.16 ± 0.03 ^a^	5.42 ± 0.25 ^a^	11.46 ± 0.50 ^a^	0.19 ± 0.05 ^a^	6.64 ± 0.00 ^a^
MHO	39.76 ± 1.34 ^a^	0.97 ± 0.06 ^a^	6.10 ± 0.54 ^a^	11.65 ± 0.08 ^a^	0.19 ± 0.09 ^a^	6.64 ± 0.00 ^a^

Mean values were given in columns for each sample and the ones which followed by different letters are significantly different (*p* < 0.05). (Abbreviations are: M 12% milk fat, H 12% hazelnut oil, O 12% olive oil, MH 6% milk fat-6% hazelnut oil, MO 6% milk fat-6% olive oil, HO 6% hazelnut oil-6% olive oil, MHO 4% milk fat-4% hazelnut oil-4% olive oil).

**Table 3:** Color values of seven types of ice creams.

Ice Cream	L*	a*	b*
M	88.72 ± 1.70 ^a^	−2.87 ± 0.45 ^a^	9.47 ± 1.23 ^a,b^
H	89.70 ± 1.62 ^a^	−2.98 ± 0.20 ^a^	8.70 ± 0.37 ^b^
O	88.24 ± 0.30 ^a^	−3.37 ± 0.35 ^a^	9.71 ± 0.27 ^a,b^
MH	88.05 ± 2.75 ^a^	−3.36 ± 0.31 ^a^	10.02 ± 0.48 ^a,b^
MO	88.48 ± 3.31 ^a^	−3.09 ± 0.45 ^a^	9.53 ± 0.75 ^a,b^
HO	88.85 ± 3.51 ^a^	−3.48 ± 0.47 ^a^	10.46 ± 1.05 ^a^
MHO	85.52 ± 3.73 ^a^	−3.42 ± 0.47 ^a^	10.05 ± 0.53 ^a,b^

Mean values were given in columns for each sample and the ones which followed by different letters are significantly different (*p* < 0.05) (Abbreviations are: M 12% milk fat, H 12% hazelnut oil, O 12% olive oil, MH 6% milk fat-6% hazelnut oil, MO 6% milk fat-6% olive oil, HO 6% hazelnut oil-6% olive oil, MHO 4% milk fat-4% hazelnut oil-4% olive oil).

**Table 4:** Sensory characteristics of ice creams supplemented with different fat type during 90-day storage period evaluated by the sensory panel.

Ice Cream	Store Days	Color and Appearance	Body and Texture	Flavour and Smell	Total Acceptability
M	1	4.53 ± 0.30 ^a^	4.23 ± 0.60 ^a^	4.33 ± 0.64 ^a^	13.03 ± 1.40 ^a,b^
	45	4.33 ± 0.50 ^a^	4.13 ± 0.57 ^a^	4.53 ± 0.63 ^a^	13.00 ± 1.03 ^a^
	90	4.13 ± 0.15 ^a^	4.00 ± 0.30 ^a^	4.26 ± 0.75 ^a^	12.40 ± 0.78 ^a^
H	1	4.46 ± 0.15 ^a^	4.63 ± 0.28 ^a^	4.36 ± 0.40 ^a^	13.46 ± 0.58 ^a^
	45	4.56 ± 0.20 ^a^	4.36 ± 0.51 ^a^	4.50 ± 0.26 ^a^	13.43 ± 0.66 ^a^
	90	4.00 ± 0.17 ^a^	4.23 ± 0.20 ^a^	4.03 ± 0.15 ^a^	12.26 ± 0.41 ^a^
O	1	4.46 ± 0.05 ^a^	4.30 ± 0.45 ^a^	3.13 ± 0.11 ^c^	11.90 ± 0.55 ^b^
	45	4.26 ± 0.30 ^a^	4.33 ± 0.50 ^a^	3.70 ± 0.10 ^b^	12.30 ± 0.75 ^a^
	90	4.10 ± 0.26 ^a^	4.43 ± 0.35 ^a^	3.46 ± 0.60 ^a^	12.00 ± 1.21 ^a^
MH	1	4.26 ± 0.30 ^a^	4.10 ± 0.36 ^a^	3.53 ± 0.32 ^b,c^	11.90 ± 0.43 ^b^
	45	4.43 ± 0.20 ^a^	4.70 ± 0.36 ^a^	4.43 ± 0.63 ^a,b^	13.56 ± 1.18 ^a^
	90	4.36 ± 0.58 ^a^	4.50 ± 0.45 ^a^	3.90 ± 0.95 ^a^	12.76 ± 1.96 ^a^
MO	1	4.43 ± 0.28 ^a^	4.46 ± 0.35 ^a^	4.33 ± 0.30 ^a^	13.23 ± 0.51 ^a,b^
	45	4.76 ± 0.25 ^a^	4.40 ± 0.30 ^a^	4.56 ± 0.20 ^a^	13.73 ± 0.55 ^a^
	90	4.20 ± 0.60 ^a^	4.40 ± 0.62 ^a^	4.63 ± 0.63 ^a^	13.23 ± 1.81 ^a^
HO	1	4.13 ± 0.64 ^a^	4.23 ± 0.47 ^a^	3.70 ± 0.1 ^a,b,c^	12.06 ± 1.05 ^a,b^
	45	4.50 ± 0.26 ^a^	4.46 ± 0.32 ^a^	4.13 ± 0.55 ^a,b^	13.10 ± 0.96 ^a^
	90	4.33 ± 0.20 ^a^	4.46 ± 0.20 ^a^	4.10 ± 0.95 ^a^	12.90 ± 1.32 ^a^
MHO	1	4.46 ± 0.15 ^a^	4.60 ± 0.20 ^a^	4.00 ± 0.10 ^a,b^	13.06 ± 0.05 ^a,b^
	45	4.20 ± 0.20 ^a^	4.03 ± 0.58 ^a^	4.53 ± 0.45 ^a^	12.76 ± 0.75 ^a^
	90	3.90 ± 0.26 ^a^	4.10 ± 0.43 ^a^	4.13 ± 0.61 ^a^	12.13 ± 1.00 ^a^

Mean values were given in columns for each sample and the ones which followed by different letters are significantly different (*p* < 0.05). (Abbreviations are: M 12% milk fat, H 12% hazelnut oil, O 12% olive oil, MH 6% milk fat-6% hazelnut oil, MO 6% milk fat-6% olive oil, HO 6% hazelnut oil-6% olive oil, MHO 4% milk fat-4% hazelnut oil-4% olive oil).
